# Editorial: Immune barrier, viral sanctuaries, and sexual transmission in the male reproductive system

**DOI:** 10.3389/fimmu.2023.1139520

**Published:** 2023-02-07

**Authors:** Jing Zhang, Wei Lu, C. Yan Cheng, Daishu Han

**Affiliations:** ^1^ Institute of Basic Medical Sciences, Chinese Academy of Medical Sciences and Peking Union Medical College, Beijing, China; ^2^ Institut de Recherche sur les Vaccins, Université Sorbonne Paris Cité, Paris, France; ^3^ Center for Biomedical Research, Population Council, New York, NY, United States

**Keywords:** testis, immune privilege, blood-testis barrier, viral reservoir, sexual transmission

The male reproductive system (MRS) is relatively isolated from other systems of the body considering its anatomical position and highly organized structure (**Figure 1**). The testis adopts an immune-privileged environment to protect immunogenic male germ cells (MGCs) from autoimmune responses. However, the immune-privileged status can be hijacked by viruses as sanctuaries to escape from systemic immune surveillance. A large spectrum of viruses has a tropism for the testis and may also infect other organs of the MRS (**Figure 1**). Viral infection of the MRS potentially impairs male fertility and leads to sexual transmission of viruses. The MRS employs several local antiviral mechanisms to reduce viral impairment of fertility and sexual transmission. Mechanisms underlying immune privilege, viral sanctuaries, and antiviral systems are noteworthy.

**Figure 1:**
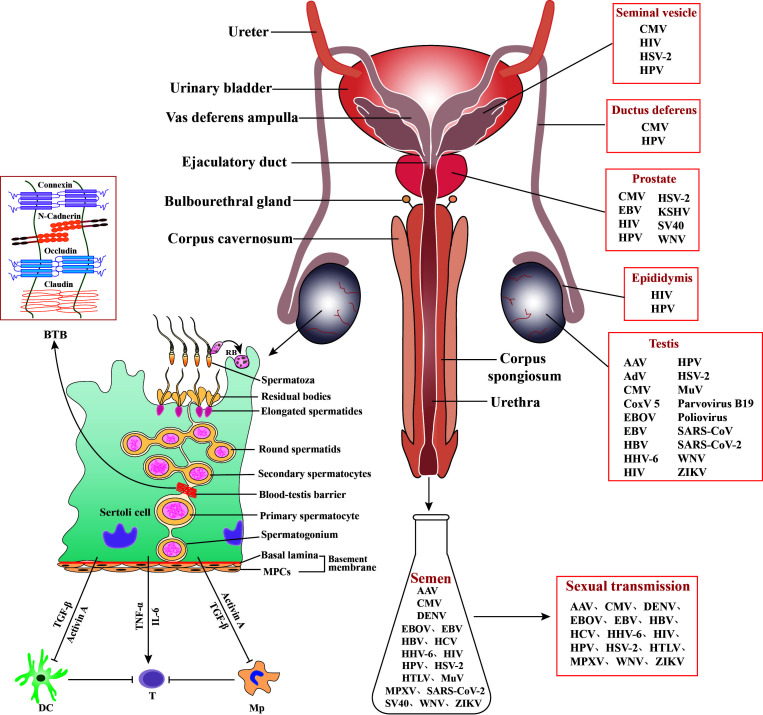
Role of Sertoli cells (SCs) in regulating testicular immunity and virus distribution in the male reproductive system (MRS). Middle panels: The MRS is composed of various organs, including testes, epididymides, prostates, seminal vesicles, and bulbourethral gland. These organs are connected by genital ducts, including ductus deferens, vas deferens ampulla, ejaculatory duct, and urethra. Left panels: The blood-testis barrier (BTB) is formed by adjacent SCs with several cellular junction proteins and separates the majority of germ cells within the adluminal compartments behind the BTB from immune cells in the interstitial spaces. SCs secrete activin A and transforming growth factor β (TGF−β) that inhibit immune responses of dendritic cells (DCs) and macrophages (Mp), thereby inhibiting effective T cell activation. SCs may also produce inflammatory factors, such as tumor necrosis factor α (TNF−α) and interleukin 6 (IL−6), in response to antigen challenges, which can activate T cells and induce inflammatory conditions. MPCs, myoid peritubular cells; RB, residual body; ⟂, inhibition; →, activation. Right panels: A broad spectrum of viruses has been detected in the MRS and in semen, and some of the seminal viruses can be sexually transmitted. AAV, Adeno-associated virus; AdV, Adenovirus; CMV, Cytomegalovirus; CoxV 5, Coxsackie virus 5; DENV, Dengue virus; EBV, Epstein-Barr virus; EBOV, Ebola virus; HBV, Hepatitis B virus; HCV, Hepatitis C virus; HHV-6, Human herpes virus 6; HIV, human immunodeficiency virus; HPV, human papillomavirus; HSV, Herpes simplex virus; HTLV, Human T lymphotropic virus; KSHV, Kaposi sarcoma-associated herpes virus; MuV, mumps virus; MPXV, Monkeypox virus. SARS-CoV, severe acute respiratory syndrome associated-coronavirus; SV40, Simian virus 40; WNV, West-Nile virus; ZIKV, Zika virus.

A large number of viruses have been detected in human semen, and some of them can exist for a prolonged period, suggesting that the MRS can harbor viral reservoirs (1). Research has focused on the testis as a place for viral sanctuaries due to its immune−privileged status. MGCs, particularly spermatozoa, can be viral carriers leading to sexual transmission. A study from Kuassivi et al. demonstrated that MGCs lack an antiviral response, thereby favoring Zika virus (ZIKV) replication and persistence. Accordingly, ZIKV and some other viruses, including hepatitis B and C (HBV and HCV), herpes simplex virus (HSV), and human papillomavirus (HPV), may be transmitted *via* spermatozoa (1). Therefore, MGCs may serve as reservoirs for some viruses and mediate sexual transmission, which is worthy of great attention.

MGCs are also considered potential viral sanctuaries because the majority of germ cells reside behind the blood−testis barrier (BTB). The BTB efficiently sequesters these germ cells within the adluminal compartments of the seminiferous tubules and immune components in the interstitial spaces. Therefore, the BTB plays a crucial role in maintaining testicular immune privilege (Li et al.). As an immune barrier, the BTB may also help viruses to escape from immune surveillance within the adluminal compartments. MGCs that are localized behind the BTB can be predominantly hijacked by viruses to serve as viral sanctuaries. The biology and regulation of the BTB have been intensively investigated (2). Yang et al. elucidated the role of the FYN non-receptor tyrosine kinase in regulating BTB integrity and permeability, which provided novel insights into the regulatory mechanisms underlying BTB function. Since FYN facilitates virus entry across the epithelial tight junction, it potentially promotes virus access to the adluminal compartments across the BTB by regulating BTB permeability. A potential role of the BTB in protecting viruses from immune surveillance remains elusive.

Sertoli cells (SCs) play a key role in regulating the immune environment of the testis through multiple mechanisms (3). In addition to the formation of the BTB by adjacent SCs, these cells also phagocytize apoptotic germ cells and secrete immune inhibitory substances. These functions of SCs are critical to prevent autoimmune responses against germ cell antigens. However, SCs also produce inflammatory cytokines in response to allo- or auto-antigen challenges, which may facilitate inflammatory conditions in the testis. Washburn et al. comprehensively reviewed the double-edged sword role of SCs in regulating the immunological pathophysiology of the testis and described the dual functions of SCs in inhibiting autoimmune responses and inducing inflammatory responses against microbial infections.

While a large spectrum of viruses infects most organs of the MRS, only a few viruses severely impair male fertility. These observations suggest that the MRS adopts antiviral mechanisms to reduce the viral impairment of fertility. Viral infection of different organs may result in distinct consequences associated with innate antiviral responses in individual organs. For example, the mumps virus (MuV) has a high tropism for the testis and efficiently replicates in testicular cells, thereby inducing orchitis that may result in infertility (Wu et al.). However, the MuV rarely infects prostatic epithelial cells, and type 1 interferon signaling restricts MuV replication in these cells (4). Human immunodeficiency virus 1 (HIV-1) and ZIKV shedding in semen can originate from different organs of the MRS (1), suggesting that multiple organs can be viral reservoirs. In addition to MGCs, other specific cells that can become viral sanctuaries in the MRS are worthy of further identification. 

A large number of virus types have been found in semen, and half of these viruses can be sexually transmitted (**Figure 1**). The sexual transmission efficiencies of viruses are extremely low, suggesting that inhibitory mechanisms underlying sexual transmission exist (5). Understanding the antiviral mechanisms in the MRS can aid in the development of strategies for the prevention of viral infection and sexual transmission. Wang et al. reviewed the viral tropism for the testis and the antiviral mechanisms in the MRS. Recent studies on the innate immune responses of the testis, epididymis, seminal vesicle, and prostate have improved our understanding of the antiviral mechanisms in the MRS. As a viral vector, semen impacts viral infection and transmission. Several seminal components, including amyloid fibrils, fibronectin, complement molecules, and TGF−β, can facilitate infection of HIV and Ebola virus (6, 7). On the contrary, other studies demonstrate that seminal exosomes inhibit infection of HIV-1 and ZIKV (8, 9), and human seminal plasma inhibits cytomegalovirus infection (10). A recent study characterized pan-antiviral activities against MuV, HSV-1 adenovirus 5, and dengue virus 2 in prostate fluids (Chen et al.). The specific antiviral factors in prostate fluids are worthy of further clarification. Discrepancies regarding the effect of semen on viral infection remain unclear.

This Research Topic covers the immune status of the testis, viral sanctuaries, and antiviral mechanisms in the MRS, as well as regulation of the sexual transmission of viruses. The contents of the Research Topic provide insights into the above issues and suggest directions for future investigation. Viral reservoirs in the MRS and antiviral factors in semen are particularly interesting for further investigation.

## Author contributions

JZ and DH drafted the editorial, and WL and CC contributed to the final version. All authors approved it for publication.
